# Opposite correlations between cation disordering and amorphization resistance in spinels versus pyrochlores

**DOI:** 10.1038/ncomms9750

**Published:** 2015-10-29

**Authors:** Blas Pedro Uberuaga, Ming Tang, Chao Jiang, James A. Valdez, Roger Smith, Yongqiang Wang, Kurt E. Sickafus

**Affiliations:** 1 Materials Science and Technology Division, Los Alamos National Laboratory, Los Alamos, New Mexico 87545, USA; 2 Thermo-Calc Software Inc., Pittsburgh, Pennsylvania 15317, USA; 3 Department of Mathematical Sciences, Loughborough University, Loughborough LE11 3TU, UK; 4 Department of Materials Science and Engineering, University of Tennessee, Knoxville, Tennessee 37996, USA

## Abstract

Understanding and predicting radiation damage evolution in complex materials is crucial for developing next-generation nuclear energy sources. Here, using a combination of ion beam irradiation, transmission electron microscopy and X-ray diffraction, we show that, contrary to the behaviour observed in pyrochlores, the amorphization resistance of spinel compounds correlates directly with the energy to disorder the structure. Using a combination of atomistic simulation techniques, we ascribe this behaviour to structural defects on the cation sublattice that are present in spinel but not in pyrochlore. Specifically, because of these structural defects, there are kinetic pathways for the relaxation of disorder in spinel that are absent in pyrochlore. This leads to a direct correlation between amorphization resistance and disordering energetics in spinel, the opposite of that observed in pyrochlores. These results provide new insight into the origins of amorphization resistance in complex oxides beyond fluorite derivatives.

## Abstract (web summary)

Radiation can cause the amorphization of crystalline materials, which needs to be understood if they are to be used in nuclear reactors. Here, the authors show a direct correlation between amorphization resistance and disordering energetics in spinel crystals, the opposite of that observed in pyrochlores.

## Graphical abstract

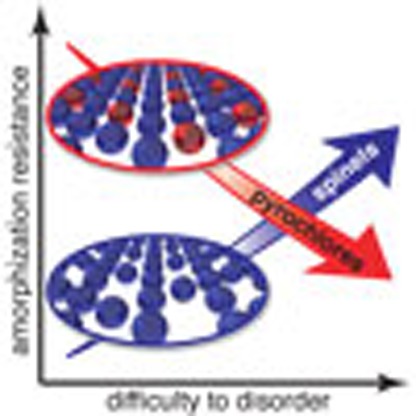

As world-wide demand for energy continues to increase, the need for energy sources that are free of green house gas emissions becomes even more pressing. Probably, the most successful such energy source is provided by nuclear energy, generating some 14% of the world's electric energy^1^. Despite this success, there is even greater potential for nuclear power, given that some 94–96% of the fuel is not utilized during typical operation^2^. This is a consequence of the fact that understanding and predicting material evolution at the extreme conditions encountered in the reactor is challenging. A primary hurdle for fusion reactors is also related to material durability under operation. There is thus a great impetus to both understand and predict radiation damage evolution in materials to enhance the capability of both current and next-generation nuclear reactors.

Oxides are of primary importance for nuclear energy production. Not only do they constitute the primary fuel form in fission reactors, but they have been proposed for a multitude of other nuclear-energy-related applications, including as nuclear waste forms, inert matrix fuels and radio-frequency windows for fusion reactors. They are also a critical component of oxide dispersion strengthened steels^3^. To realize the promise of these materials, they must be optimized via crystal structure and chemistry to ensure maximum performance. There has thus been immense work on understanding the origin of radiation tolerance in these materials, particularly the susceptibility to amorphization. For example, previous work^4^,^5^ has shown a correlation between the amorphization resistance of pyrochlores (A_2_B_2_O_7_) and the ability of the cation sublattice to disorder (A_A_+B_B_→A_B_+B_A_). As the energy cost for disordering increases with changing A or B cation chemistry, the tendency of the pyrochlore to amorphize also increases. The general trend regarding the order-to-disorder transformation has been shown to hold in other fluorite derivatives (oxides with a structure related to fluorite) such as the so-called *δ*-phase compounds (for example, A_4_B_3_O_12_ and A_6_B_1_O_12_)^5^. Pyrochlores have received extensive attention as they have natural analogues for the encapsulation of radionuclides^6^.

Although the original work connecting amorphization resistance to cation disordering energetics in pyrochlore relied on empirical potentials to establish that correlation^4^,^5^, the physical trends identified have been validated using density functional theory (DFT) calculations, which have examined both the energetics to create antisite pairs^7^,^8^ and to fully disorder pyrochlore to form disordered fluorite^7^,^8^,^9^. These calculations show remarkable agreement with experiment. First, calculations of the energetics to fully disorder pyrochlore correlate well with observed order-to-disorder transition temperatures from the phase diagrams of several pyrochlore chemistries^9^. Second, all of the calculations, including those using the original empirical potential^10^, find non-monotonic behaviour for the titanate family of pyrochlores, with compounds having A cation composition at or near A=Gd exhibiting the highest energy to disorder. This is precisely the composition at which experimental observations find the highest critical amorphization temperature^11^, indicating it is the easiest to amorphize. Thus, there is a strong correlation between the energetics of cation disorder and amorphization susceptibility in pyrochlores. Other factors have also been identified as correlating with the radiation tolerance of these compounds, including the enthalpy of formation of the pyrochlore^12^ and the ionic versus covalent nature of the chemical bonds^13^,^14^. These properties are related to the ability of the material to disorder. In particular, bonds that are more ionic in nature can be rearranged more easily than covalent bonds. The disordering energy, although not as fundamental as the nature of the bond, is a more convenient measure as it can be estimated from the phase diagram^5^ and can be readily calculated^8^,^9^. Finally, this correlation between higher energetics for cation disorder and ease of amorphization has been observed in other materials, including other fluorite derivatives^5^,^15^,^16^ and even ordered intermetallics^17^,^18^,^19^,^20^, where the correlation has received significant attention. All of these compounds have in common the fact that the metal sublattices are fully dense; there are no structural vacancies.

That said, cation disordering alone does not completely predict trends in amorphization behaviour under irradiation. Pyrochlores with similar disordering energetics do exhibit significantly different amorphization resistance. For example, Er_2_Ti_2_O_7_ and Nd_2_Zr_2_O_7_ have similar energetics for disordering^9^, but while Er_2_Ti_2_O_7_ can be easily amorphized^4^, Nd_2_Zr_2_O_7_ can only be amorphized with high-energy ions^21^. This is epitomized by the Sn pyrochlores, which have extremely high disordering energies^9^, but are often very resistant to amorphization^22^. An even better predictor of amorphization resistance is the gap in energy between the disordered state and the amorphous state, which is qualitatively indicated by the extent of stability of the disordered phase in the phase diagram^5^. However, the melting temperature or the free energy of the amorphous phase is challenging to estimate theoretically and is often not available from experiment. Thus, the disordering energetics, although an incomplete heuristic, is still valuable for identifying compounds that resist amorphization. It is such a strong indicator of amorphization susceptibility for the titanate pyrochlores as, in those systems, the pyrochlore stabilty field extends to the melting temperature.

Radiation damage behaviour in spinels has also been extensively studied, with most work focused on MgAl_2_O_4_. Experiments have shown that MgAl_2_O_4_ is very radiation tolerant, with one primary consequence of the damage being disordering of the cation sublattice^23^,^24^. This cation disorder can lead to order-to-disorder transformations, which have been observed under neutron irradiation^23^ and swift heavy ion irradiations^24^,^25^, where the energy loss is primarily due to electronic stopping, whereas a rocksalt phase can form under conditions where nuclear stopping dominates^26^. In addition, under certain conditions, dislocation loops have been observed to form^27^, although these loops are not able to grow to extremely large sizes, preventing subsequent void swelling^28^,^29^. Finally, although MgAl_2_O_4_ is considered to be a ‘radiation-resistant' material with respect to swelling and amorphization, it can also be amorphized under irradiation, more typically with heavy ions at low temperatures^30^,^31^,^32^. Other spinels have also been examined, including ZnAl_2_O_4_ (ref. 25), MgCr_2_O_4_ (ref. 25), MgGa_2_O_4_ (ref. 33), FeCr_2_O_4_ (ref. 34) and Mg_2_SnO_4_ (ref. 35). These studies see significant differences in the radiation damage response of spinels as a function of chemistry. However, despite this large body of work, there has been no correlation between that response and the fundamental properties of each spinel. Interestingly, the first complex oxide in which cation disordering was identified as a key factor in the radiation tolerance of the compound was MgAl_2_O_4_ spinel^23^,^36^.

Here we show that the correlation between cation disordering energetics and amorphization resistance found in pyrochlores does not hold in spinels (AB_2_O_4_), which have a crystal structure related to rocksalt. In fact, the opposite trend occurs, with spinels that are more difficult to disorder exhibiting higher resistance to amorphization. We show that this is related to the structure of the cation sublattice and how that structure is fundamentally different in spinels versus pyrochlores (and other fluorite derivative oxides and intermetallics). In particular, inherent structural vacancies that exist on the cation sublattice of spinel (relative to rocksalt) allow for relaxation mechanisms under irradiation that do not exist in fluorite derivatives. That is, the high activation barrier for the reverse disorder-to-order transformation that exists for pyrochlores does not exist for spinels. Together, the contrasting behaviour of spinels and pyrochlores under irradiation lead to a generalized view of the amorphization response of complex oxides to irradiation.

## Results

### Characterization of irradiated spinels

**Figure 1:**
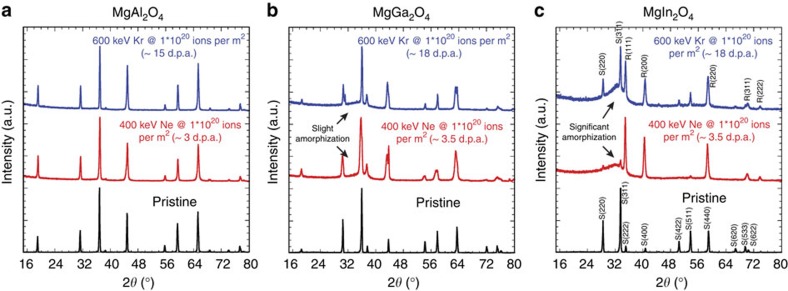
GIXRD characterization of irradiated spinels. (**a**) MgAl_2_O_4_, (**b**) MgGa_2_O_4_ and (**c**) MgIn_2_O_4_ in the pristine as-synthesized state (black curves), after irradiation to a fluence of 10^20^ Ne ions per m^2^ (red curves) and after irradiation to a fluence of 10^20^ Kr ions per m^2^ (blue curves). MgAl_2_O_4_ remains unchanged after these irradiation fluences. MgGa_2_O_4_ shows indications of amorphization and the reduction in diffraction peaks associated with the spinel structure. These trends are even more apparent in the MgIn_2_O_4_ spinel in which there is significant amorphization and the spinel diffraction peaks are almost completely absent after irradiation. The spinel and rocksalt peaks are labelled S and R, respectively, for MgIn_2_O_4_ for reference.

Figure 1 shows grazing incident X-ray diffraction (GIXRD) results for three different spinels for three different conditions: as synthesized (pristine), irradiated to a fluence of 10^20^ Ne ions per m^2^ (corresponding to damage levels of roughly 3–4 d.p.a., as determined from SRIM (Stopping and Range of Ions in Matter) calculations^37^), and irradiated to a fluence of 10^20^ Kr ions per m^2^ (15–20 displacements per atom (d.p.a.)). These two irradiation conditions were chosen to provide insight into the role of spectrum effects on the relative stability of the three spinels. In particular, the electronic versus nuclear stopping is significantly different for these two conditions. In the case of Ne, electronic stopping is greater than nuclear stopping, being about six times as great at the surface and decaying to a ratio of roughly one at half a micrometre. In contrast, nuclear stopping is greater for Kr, with the electronic/nuclear stopping ratio being 0.4 or less throughout the irradiated region. Thus, these two irradiation conditions probe the relative importance of electronic versus nuclear effects in amorphizing the compounds. All irradiations were performed at *T*=100 K. The pristine diffraction patterns confirm that all three materials are spinel in the as-synthesized state. After irradiation, the evolution of each spinel depends on the chemistry of the material. MgAl_2_O_4_ shows no indication of amorphization. In contrast, MgIn_2_O_4_ shows significant amorphization. MgGa_2_O_4_ lies somewhere in between. This trend occurs for both irradiation conditions, suggesting that the fundamental behaviour is insensitive to irradiation spectrum, although the details certainly are. We note that we also performed irradiations with 120 MeV Au ions, which have an electronic stopping of greater than 20 keV nm^−1^, at room temperature and observed no amorphization in any of these spinels, indicating the strong dependence of the response of the material to the irradiation type.

**Figure 2:**
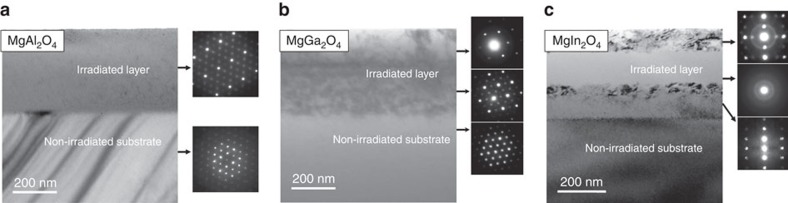
Microscopy of irradiated spinels. Cross-sectional TEM images and corresponding selected area electron diffraction (SAED) patterns of irradiated (**a**) MgAl_2_O_4_, (**b**) MgGa_2_O_4_ and (**c**) MgIn_2_O_4_. In the case of MgAl_2_O_4_, the SAED pattern of the irradiated layer shows that the material is still fully crystalline. In contrast, the irradiated layer in MgGa_2_O_4_ is partially amorphized and the most damaged layer in MgIn_2_O_4_ is completely amorphized after irradiation to the same fluence (600 keV Kr at a fluence of 10^20^ ions per m^2^ at 100 K).

The interpretation of the GIXRD measurements are corroborated by the cross-sectional transmission electron microscopy (TEM) images in Fig. 2. The irradiation conditions for all cases shown was 600 keV Kr to a fluence of 10^20^ ions per m^2^ at 100 K. In MgAl_2_O_4_, the irradiated layer remains fully crystalline. In MgGa_2_O_4_, there is partial amorphization in the irradiated layer, but it remains mostly crystalline. In contrast, in MgIn_2_O_4_, the peak damage region is fully amorphized while the the top portion of the irradiated layer has partially amorphized. We note that while the irradiation was performed at 100 K and the GIXRD and TEM at 300 K, we expect no significant change in amorphous content as recrystallization of amorphous spinel occurs only at temperatures in excess of 900 K (ref. 38).

### Irradiation-induced defects and disordering in spinels

**Figure 3:**
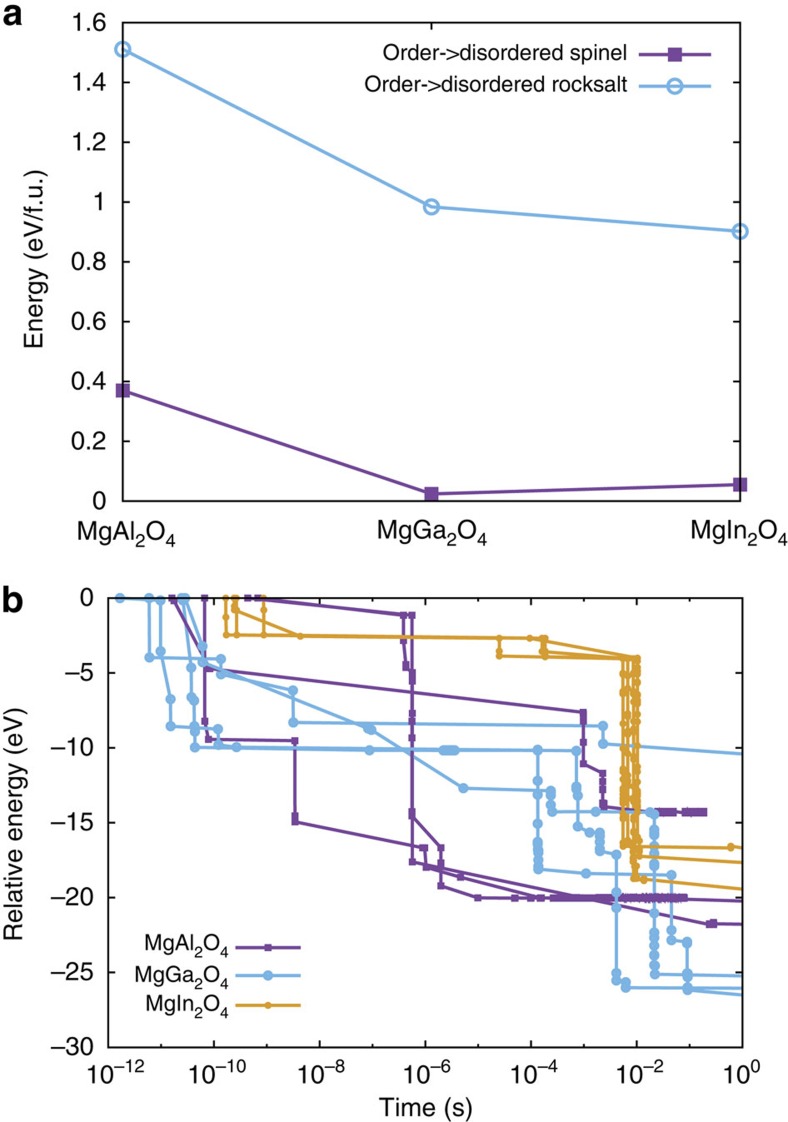
Thermodynamic and kinetic behaviour in spinels. (**a**) Relative energy, in eV per formula unit, of disordered spinel (purple curve/filled symbols) and defective rocksalt (blue curve/open symbols) for MgAl_2_O_4_, MgGa_2_O_4_ and MgIn_2_O_4_, as calculated using SQS and DFT. The energetics of the ordered spinel to disordered spinel transformation do not completely track the amorphization susceptibility observed in the experiments. The energetics of disordering all the way to rocksalt follow the same trend as the experiments. (**b**) TAD simulations showing the evolution of MgAl_2_O_4_ (purple curve/squares), MgGa_2_O_4_ (blue curve/open circles) and MgIn_2_O_4_ (yellow curve/filled circles), initially placed in a defective rocksalt structure. The zero of energy is the initial defective rocksalt structure, in which the atomic positions are minimized but the cell dimensions are held at those for ordered spinel. For each compound, four different simulations, starting from the same structure but evolved with different random number seeds, are shown. The points represent transitions between states that occur at the indicated times. The lines are guides for the eye. There is a general trend that relaxation is fastest for MgAl_2_O_4_ and slowest for MgIn_2_O_4_.

It is well established, from both experiment and theory, that MgAl_2_O_4_ is a normal spinel, with a small amount of inversion *i* (refs 39, 40; mixing of the A and B cation sublattices, defined as the fraction of A sites containing B cations; *i*∼0 for natural spinel and ∼0.2 for synthetic spinel), MgGa_2_O_4_ is a random spinel with *i=*∼0.67–0.9 (refs 41, 42, 43, 44) and MgIn_2_O_4_ is an inverse spinel with *i* approaching 1 (ref. 41). Indeed, as revealed by DFT calculations of the energy to form a disordered/random spinel (Fig. 3a), MgAl_2_O_4_ is significantly harder to disorder than the other two spinels, and MgGa_2_O_4_ is the easiest (similar results were reported in ref. 45). Based on the insights gained from the pyrochlore studies, this suggests that the Al spinel should amorphize most readily and the In spinel should be the most difficult to amorphize. Clearly, the results in Fig. 1 reveal the opposite trend. In spinels, simply disordering the cation sublattice does not correlate with amorphization resistance.

Under irradiation, point defects are inevitably formed. In complex oxides, the general defect reaction that occurs under irradiation can be summarized as


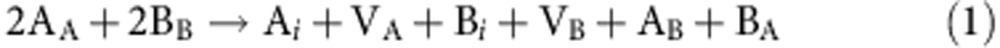



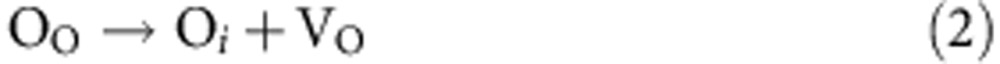


In pyrochlores, the cation interstitials and vacancies have a tendency to transform to antisite defects (either thermally or directly in-cascade^46^), leading to a simplified cation reaction of


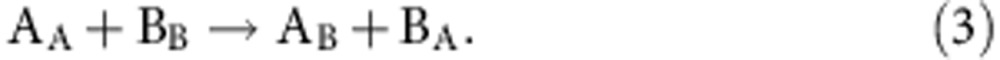


As discussed in ref. 5, combined with the oxygen defect reaction in equation 2, this reaction, when applied *ad infinitum*, transforms the pyrochlore structure to a defective fluorite structure. Ample experimental evidence indicates that this transformation indeed occurs in pyrochlores under irradiation^4^,^5^,^47^,^48^,^49^,^50^,^51^.

**Figure 4:**
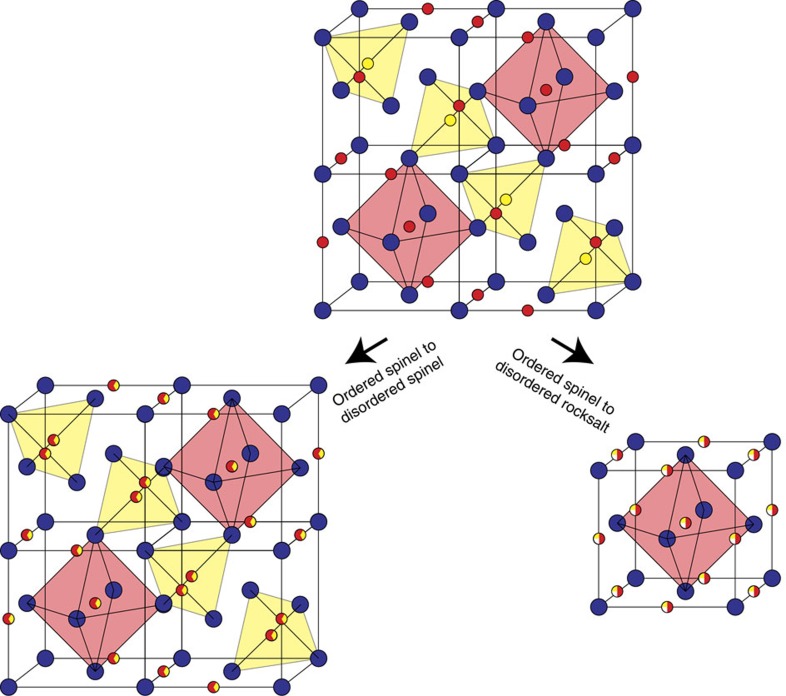
Disordering transformations in spinel. Schematic showing the structure of spinel and the structural modifications that occur when ordered spinel is transformed to disordered spinel and to disordered rocksalt, as described by equations 2 and 1, respectively. Although the first involves simply swapping cations, the second transformation requires a movement of cations from tetrahedral sites (highlighted by the yellow tetrahedra) to empty octahedral sites. Blue spheres represent oxygen while yellow spheres are Mg and red spheres are B cations. The mixed spheres indicate sites with mixed (random) populations. Figure inspired by ref. 66.

In contrast, in spinels, the cation interstitials and vacancies are relatively stable. This is a consequence of the structural defects in spinel, relative to the basic structure of rocksalt. (Here, following ref. 52, we use the term *basic structure* to refer to the structure from which other crystal structures are derived. Thus, rocksalt is the basic structure of spinel and fluorite is the basic structure of pyrochlore.) Just as pyrochlore can be conceptualized as a fluorite derivative compound, spinel can be considered a rocksalt derivative and, similar to the order-to-disorder transformation observed in irradiated pyrochlore, irradiated spinels transform into a rocksalt structure^26^,^30^,^53^. This is evident in Fig. 1c which shows that MgIn_2_O_4_ loses spinel superlattice peaks but retains rocksalt peaks under irradiation, indicating the transformation to a rocksalt structure. Thus, under irradiation, the order-to-disorder transformation in spinels that is analogous to the pyrochlore-to-defective fluorite transformation is not ordered spinel-to-disordered spinel (as would be described by equation 3) but rather spinel-to-defective rocksalt. That is, the generation of defects as described by equation 1 necessarily transforms spinel into defective rocksalt. These reactions are schematically illustrated in Fig. 4. We note that, in this view, disordered spinel is not equivalent to disordered rocksalt, but disordered pyrochlore and disordered fluorite are equivalent. The difference is whether cation identities are simply changed or whether cations must be also be moved to interstices to describe the associated disordering process.

As discussed, in pyrochlores, amorphization resistance is inversely correlated with the energy to disorder the material. We find that the opposite is true in spinels. Figure 3a shows that the thermodynamic energy to transform spinel into defective rocksalt is highest for Al spinel, lowest for In spinel and intermediate for Ga spinel. These energies correlate directly with the amorphization transformations described in Fig. 1. The reason there is a direct correlation between amorphization resistance and disordering energy in spinel but an inverse correlation in pyrochlore is due to the structural cation defects present in spinel that are not present in pyrochlore.

### Consequences of structural cation vacancies

If one considers the structure of the defective fluorite phase that forms in irradiated pyrochlores, it contains oxygen vacancies relative to perfect fluorite, but the cation sublattice is fully dense. For every cation in fluorite, there is a cation in pyrochlore. In contrast, the defective rocksalt phase formed in spinels has a fully dense oxygen sublattice, but vacancies on the cation sublattice, relative to perfect rocksalt. We propose that these vacancies provide relaxation pathways for local reordering of the cation sublattice. Further, the tendency for reordering is greatest in those spinels in which the energy of the defective rocksalt phase is highest; there is a larger energy gain in reordering such spinels and thus the kinetic pathways for reordering are faster. This explains why the Al spinel is most resistant to amorphization and the In spinel is least resistant to amorphization.

In support of this hypothesis, we provide two more pieces of evidence. First, in molecular dynamics (MD) simulations of collision cascades in the three spinels^54^, we observed that more rocksalt-like defects formed in In spinels than in either Al or Ga spinels. This indicates that rocksalt-like defects are more stable in In spinels, consistent with the lower energy of the rocksalt phase as found via DFT in Fig. 3a. More specifically, the spinel-to-rocksalt transformation requires the transfer of A (Mg) or B (Al, Ga, In) cations from occupied 8*a* sites (in Wyckoff notation) to empty 16*c* sites, as well as mixing between A and B cationic species on both 16*c* and 16*d* sites. The MD simulations reveal both that a greater number of defects is formed in In spinel as compared with the other two spinels and that a greater shift of ions from 8*a* to 16*c* sites occurs in In than either Al or Ga spinels. That is, rocksalt-like defects which may form during the thermal spike are not as stable in the Al compound as compared with the In compound. This structural change is not so apparent in Ga spinel as compared with Al, but the fact that more defects are formed in Ga spinel than in Al spinel suggests such rocksalt-like defects are also more stable in MgGa_2_O_4_ than in MgAl_2_O_4_. In particular, Al interstitials in MgAl_2_O_4_ readily decayed to antisites, although B cation interstitials were much more stable in the other two spinel compounds. Further, in those spinels, more extended defect structures exhibiting greater degrees of disorder were formed, although it is difficult to classify those regions as being in a rocksalt structure. Finally, in the MD simulations, we only considered isolated collision cascades; the transformation to a defective rocksalt phase may be more complete after multiple cascades.

The MD simulations indicate that the formation of a defective rocksalt in irradiated spinels is hardest when B=Al. This is because, in contrast to pyrochlore, when disordering defects form under irradiation, as they will naturally do in the thermal spike of the collision cascades, there are pathways to anneal those defects out because of the cation vacancies that are ubiquitous in the material. To test this hypothesis, we have created defective rocksalt structures of each of the three spinels and annealed them with temperature-accelerated dynamics (TAD)^55^ at 100 K, the same temperature at which the experiments were performed. Figure 3b shows the energy of each spinel as a function of time, for four realizations for each spinel to account for the stochastic nature of each trajectory. As is evident from the results, annealing progresses much more slowly in the In spinel than in the Al spinel, with Ga intermediate. In particular, after about 1 μs, the Al spinel tends to have annealed the most, whereas the In spinel has annealed the least. This shows that, even if each spinel can be placed into a defective rocksalt structure under irradiation, because of the high energy of such a phase in the Al spinel and the presence of cation vacancies to facilitate cation migration, the Al compound will relatively quickly revert back to the spinel structure, whereas this process will be much slower in MgIn_2_O_4_, as it does not have nearly as high of a thermodynamic driving force in that compound to reorder.

The faster annealing of the Al spinel is not simply a consequence of faster diffusion, as one might suspect from the smaller size of the Al ions compared with the other B cations (0.125, 0.21 and 0.22 nm for Al, Ga, and In, respectively). In fact, with these potentials, the mobility of cation defects tends to be slower in ordered Al spinels as compared with the other two spinels (A cation interstitial migration energies are 0.3 and 0.6 eV, A vacancy migration energies are 1.0 and 0.7 eV, and B cation vacancy migration energies are 0.9 and 2.0 eV, in MgIn_2_O_4_ and MgAl_2_O_4_, respectively; B cation interstitials are unstable in all of the compounds^56^). Thus, it does not seem that the faster annealing observed in the Al spinel is due to intrinsically faster defect kinetics, but is related to the higher thermodynamic driving force to reorder the compound.

## Discussion

Thus, in spinels, we find that (i) amorphization resistance correlates directly with the energy to form a defective rocksalt structure (largest for B=Al, smallest for B=In), (ii) collision cascades form more rocksalt-like defects when the defective rocksalt structure is low in energy (greater for B=In than for B=Al,Ga) and (iii) if forced into the defective rocksalt structure, recovery towards spinel is fastest for spinels in which the energy of the defective rocksalt phase is highest (fastest for B=Al, slowest for B=In). These results highlight the importance of kinetic processes in both the formation of defective rocksalt and the subsequent reversion to the spinel structure in these compounds. They further emphasize the importance of the defective rocksalt structure in understanding the relative response of these three compounds. Radiation damage drives the spinel structure towards rocksalt. If the energy of the rocksalt phase is high, there will be a higher driving force to recover the spinel structure, and the cation vacancies in the structure facilitate this recovery. If this driving force is small, the material will remain in a rocksalt-like structure, which is relatively high in energy, and eventually build up so much energy that it amorphizes.

**Figure 5:**
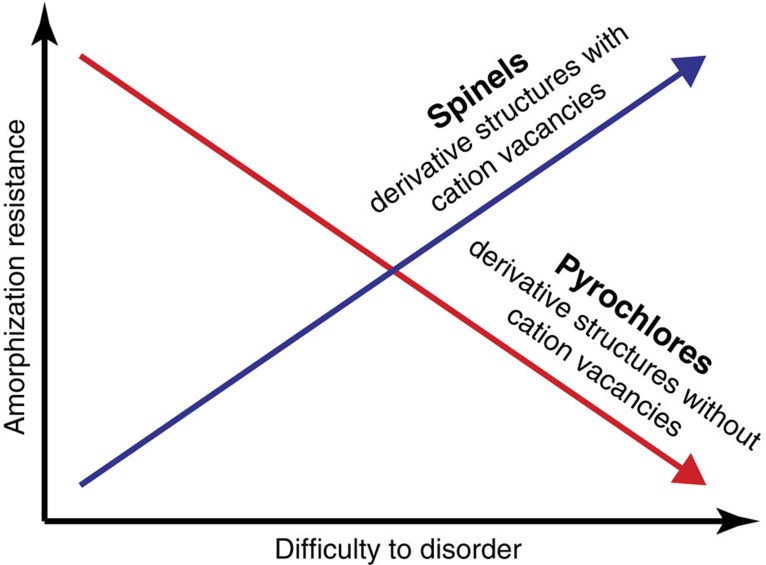
Relationship between disordering and amorphization. Schematic figure highlighting the relationship between the energetics of disordering and amorphization resistance as a function of the cation structure of the derivative compound, spinel or pyrochlore. In spinels that have cation vacancies relative to the basic rocksalt structure, amorphization resistance is proportional to the difficulty to disorder the compound, as the kinetics of reordering, facilitated by the cation vacancies, is faster as the disordered phase becomes less favourable. In contrast, in fluorite-derivative compounds that have the same cation density as the basic fluorite structure, these kinetics are absent and energy builds up fastest in compounds that have higher energies to disorder. These compounds are thus less resistant to amorphization. We propose that these concepts hold more generally to other classes of compounds as well.

Comparing the amorphization behaviour under irradiation in pyrochlores and spinels, then, in pyrochlore, amorphization resistance is inversely correlated with the energy of the defective basic structure, whereas in spinel, amorphization resistance is directly correlated with this energy. This behaviour is schematically illustrated in Fig. 5. This difference is a consequence of cation defects in the defective basic structure, which are present in the case of spinel but absent in the case of pyrochlore. Thus, reordering in spinel can occur via short-range events between these cation defects and the cations themselves. In pyrochlore, reordering must occur via the long range diffusion of radiation-induced defects, which, further, are often unstable, resulting in complicated reordering processes that occur over very long time scales^46^. In both cases, radiation damage drives the system from the initial (spinel or pyrochlore) structure to the defective basic (rocksalt or fluorite) structure. In pyrochlores, because there is no easy mechanism to recover ordering, energy builds up fastest in those compounds with the highest disordering energy. In spinels, in contrast, as there is a mechanism for reordering, recovery occurs fastest in those spinels that have the highest disordering energy. In the end, the difference between spinels and pyrochlores is due to kinetic processes involving cations that can occur in spinels but not in pyrochlores.

This suggests that one path to enhancing the amorphization resistance of complex oxides is to introduce cation vacancies through either doping or non-stoichiometry. Indeed, this very idea was examined in the case of spinel by comparing the irradiation response of MgAl_2_O_4_ (or, equivalently, MgO·Al_2_O_3_) with alternatively MgO·3(Al_2_O_3_)^57^ and MgO·2.4(Al_2_O_3_)^58^. The excess Al_2_O_3_ content leads to additional cation vacancies in the structure^59^. In the first experiment, conducted at low temperatures (100 and 300 K), the non-stoichiometric spinel performed worse under irradiation than the stoichiometric sample. However, in the second experiment, performed at 873 K, the non-stoichiometric spinel exhibited superior radiation tolerance. This suggests that (i) the cation vacancies play an important role in the radiation tolerance of these compounds and (ii) conditions must be conducive for these vacancies to be mobile. In the first experiment, the authors found that the non-stoichiometric spinel was more prone to disorder transformations than the stoichiometric sample, similar to the In spinels examined here, suggesting that the non-stoichiometric spinel is easier to disorder. Thus, in the view proposed here, the excess cation vacancies were not as strongly driven to lead to reordering, rendering the non-stoichiometric spinel less stable at lower temperatures. At higher temperatures, where the kinetics are active even when the thermodynamic driving forces for reordering are small, the vacancies are able to enhance the reordering of the compound.

Finally, we propose that, together, these results indicate a more general perspective on the amorphization susceptibility of complex oxides. In oxides with fully dense cation sublattices, such as fluorite derivatives, amorphization resistance inversely correlates with the ability of the oxide to disorder. (The same behaviour is observed in intermetallic compounds with fully dense metal sublattices.) In oxides with less-than fully dense cation sublattices, such as spinels, amorphization resistance directly correlates with disordering energy. The extent to which these relationships hold in different classes of complex oxides must still be established. Further, as discussed, cation disordering is only part of the picture; a more comprehensive view requires knowing the energetics of the amorphous phases. However, the new insights from this work provide new possibilities for discovering amorphization-resistant complex oxides for extreme environments.

## Methods

### Experiments

Polycrystalline spinel samples were synthesized by conventional ceramic processing procedures. The measured density of three spinels (MgAl_2_O_4_, MgGa_2_O_4_, MgIn_2_O_4_) were 3.38, 5.24 and 5.46 g cm^−3^, respectively, which is greater than 90% of the theoretical values for these compounds.

Ion irradiations were performed at cryogenic temperature (∼100 K) using a Danfysik High Current Research Ion Implanter operating at 200 kV in the Ion Beam Materials Laboratory at Los Alamos National Laboratory. 600 keV Kr ^+++^ and 400 keV Ne ^++^ ions were used in this study. The conversion between fluence and d.p.a. was achieved using the Monte Carlo programme SRIM (Stopping and Range of Ions in Matter)^37^, using displacement threshold energies of 40 eV for all species. Although this is an arbitrary assumption, other studies have shown that the qualitative interpretation of the damage profiles is not sensitive to the detailed choice of displacement threshold energies^60^.

Samples were examined before and after irradiation using GIXRD. GIXRD measurements were made using a Bruker AXS D8 Advanced X-ray diffractometer, Cu-K radiation, in *θ*–2*θ* geometry and at an X-ray incidence angle of 0.25°, which probes an estimated sample depth of <100 nm. Under this incidence angle, X-rays are scattered from the near-surface of these samples within a depth significantly shallower than the range of these ions (calculated ion ranges are 500 nm for Kr and 750 nm for Ne). Ion-irradiated samples were also prepared in cross-sectional geometries for TEM examination. The irradiation-induced microstructural characterization was examined using both a Philips CM-30 and a FEI Tecnai F30 electron microscope, each operating at 300 kV.

### Modelling

DFT calculations were performed using the all-electron projector augmented wave method^61^ within the local density approximation with the VASP code^62^. A plane-wave cutoff of 500 eV and dense *k*-point meshes were used to ensure convergence. The lattice parameters and all atomic positions were allowed to relax, although the cells were constrained to be cubic. Disordered spinel and defective rocksalt structures were modelled using the special quasirandom structure (SQS) approach^63^. The SQSs were generated as described in ref. 45.

The MD studies are described in the associated references. Both the MD and TAD simulations used potentials of the Buckingham form as described in ref. 54. Although these potentials do not provide the quantitative accuracy of DFT calculations, they do provide qualitative physical trends that are consistent with both experiment^4^,^5^ and DFT^64^,^65^, providing confidence in the results presented here.

TAD^55^ was used to anneal structures initially placed in a defective rocksalt structure, generated using the SQS approach and expanded 2 × 2 × 2 to fulfill minimum image requirements. The low temperature was set to 100 K (to match the irradiation conditions in the experiment), whereas the high temperature used to explore the potential energy landscape was set to 1,000 K. The same SQS structure was used for all calculations. The initial lattice constant for each compound was set to that of the corresponding ordered spinel structure at 0 K. The TAD parameters *ν*_min_ and *δ* were set to 10^12^ s^−1^ and 0.05, respectively.

## Additional information

**How to cite this article:** Uberuaga, B. P. *et al.* Opposite correlations between cation disordering and amorphization resistance in spinels versus pyrochlores. *Nat. Commun.* 6:8750 doi: 10.1038/ncomms9750 (2015).
